# Copper Nitride Nanowire Arrays—Comparison of Synthetic Approaches

**DOI:** 10.3390/ma14030603

**Published:** 2021-01-28

**Authors:** Aleksandra Scigala, Edward Szłyk, Tomasz Rerek, Marek Wiśniewski, Lukasz Skowronski, Marek Trzcinski, Robert Szczesny

**Affiliations:** 1 Faculty of Chemistry, Nicolaus Copernicus University in Toruń, Gagarina 7, 87-100 Toruń, Poland; a.scigala@doktorant.umk.pl (A.S.); eszlyk@chem.umk.pl (E.S.); marekw@umk.pl (M.W.); 2 Institute of Physics, Faculty of Physics, Astronomy and Informatics, Nicolaus Copernicus University in Toruń, Grudziadzka 5, 87-100 Torun, Poland; t.rerek@doktorant.umk.pl; 3 Institute of Mathematics and Physics, UTP University of Science and Technology, Al. Prof. S. Kaliskiego 7, 85-796 Bydgoszcz, Poland; lukasz.skowronski@utp.edu.pl (L.S.); marek.trzcinski@utp.edu.pl (M.T.)

**Keywords:** Cu_3_N, CuO, nanowires, thin films, physical vapor deposition (PVD), electroplating

## Abstract

Copper nitride nanowire arrays were synthesized by an ammonolysis reaction of copper oxide precursors grown on copper surfaces in an ammonia solution. The starting Cu films were deposited on a silicon substrate using two different methods: thermal evaporation (30 nm thickness) and electroplating (2 μm thickness). The grown CuO or CuO/Cu(OH)_2_ architectures were studied in regard to morphology and size, using electron microscopy methods (SEM, TEM). The final shape and composition of the structures were mostly affected by the concentration of the ammonia solution and time of the immersion. Needle-shaped 2–3 μm long nanostructures were formed from the electrodeposited copper films placed in a 0.033 M NH_3_ solution for 48 h, whereas for the copper films obtained by physical vapor deposition (PVD), well-aligned nano-needles were obtained after 3 h. The phase composition of the films was studied by X-ray diffraction (XRD) and selected area electron diffraction (SAED) analysis, indicating a presence of CuO and Cu(OH)_2_, as well as Cu residues. Therefore, in order to obtain a pure oxide film, the samples were thermally treated at 120–180 °C, after which the morphology of the structures remained unchanged. In the final stage of this study, Cu_3_N nanostructures were obtained by an ammonolysis reaction at 310 °C and studied by SEM, TEM, XRD, and spectroscopic methods. The fabricated PVD-derived coatings were also analyzed using a spectroscopic ellipsometry method, in order to calculate dielectric function, band gap and film thickness.

## 1. Introduction

Metal nitrides have long been known for their unique physicochemical properties which have been exploited in various applications, for example, in electronics and optics, sensors, wear-resistant coatings and catalysis [1,2,3,4]. Among this class of compounds, copper nitride (Cu_3_N) has attracted significant attention in recent years due to its application in write-once optical recording media [5,6], spintronic systems [7] and electro-catalysis [8,9]. Copper nitride is a non-toxic semiconductor (band-gap: 0.25–1.90 eV) which exhibits low reflectivity and high electrical resistivity (at room temperature) [10,11,12]. It is a metastable compound, decomposing to CuO in the presence of air and moisture [13], and to metallic copper and nitrogen upon thermal treatment [14]. Copper nitride features a specific cubic anti-ReO_3_ crystallographic structure, with a vacant center of the cell, which allows for the intercalation of foreign atoms into the structure, remarkably changing the material properties [15,16]. The specific properties of Cu_3_N can also be controlled by the different process conditions of its preparation. Until recently, research related to copper nitride mainly concerned the deposition of thin films, especially by physical methods, such as radio frequency (RF) or direct current (DC) magnetron sputtering techniques [17,18,19,20], molecular beam epitaxy (MBE) [21] or pulsed laser deposition (PLD) [22,23]. A significant increase in the number of new synthesis methods in the first decade of the 21st century was mainly related to the fabrication of Cu_3_N nanoparticles by chemical methods [24]. This allowed for the development of innovative, and potentially useful applications and possibilities for taking copper nitride as a base for new, more complex materials [8,25,26]. For example, Cu_3_N has been revised as a promising anode material, useful for rechargeable Li-ion batteries [27]. However, the fabrication of Cu_3_N thin films using chemical deposition methods is still rare, and methods described in the literature include chemical vapor deposition (CVD), where copper(II) hexafluoroacetylacetonate (Cu(hfac)_2_) and ammonia were used as a precursors [28,29], and solution-based deposition (spin- and dip-coating), followed by the ammonolysis reaction [30]. The ammonolysis reaction is the most commonly utilized pathway of binary late transition metal nitrides synthesis. In this process, a solid metal precursor reacts with gaseous ammonia and the reaction can be controlled by changing parameters such as the temperature, reaction time or ammonia gas flow rate. Ammonolysis is also the oldest approach for Cu_3_N synthesis, first being reported by Juza and Hahn in 1938, who obtained pure Cu_3_N powder from a CuF_2_ precursor [31,32]. The same copper(II) fluoride precursor was used for single-phase copper nitride by Gregory et al. [15]. Despite the known limitation of the ammonolysis reaction, resulting from the low thermal stability of Cu_3_N, this process can be considered as a convenient way for nano-structural copper nitride fabrication, and also supports other forms of materials [33]. Moreover, the useability of ammonolysis also results from the possibility of application of various precursors, such as Cu(II) salts or copper oxides. Recently, the growth of copper nitride nanowire arrays on copper foams has attracted a great deal of interest. The two-step synthesis consists of immersing the Cu foam in an alkaline solution ((NH_4_)_2_S_2_O_8_/NaOH) to form Cu(OH)_2_, and then of the ammonolysis of the hydroxide to produce nitride [34,35]. In general, solution-based synthesis is a very common, effective pathway to prepare various low-dimensional nanostructures (e.g., oxides) with large specific surface areas, which is beneficial for potential applications in catalysis or sensor designs [36,37]. The CuO nanostructures are mainly obtained from Cu(II) containing salts [36,38], but growing these structures directly from metallic copper is an alternative synthesis approach [39,40,41]. Thus, in presented work we would like to combine the advantages and simplicity of ammonolysis and the growth of copper nanowires in a solution with two different methods of initial metallic copper deposition.

In this study, we have developed a modified simple procedure for the fabrication of copper nitride nanowire arrays by the ammonolysis of copper(II) oxide films. The oxide/hydroxide precursor structures were obtained in the growth process on a copper surface under alkaline oxidative conditions, followed by calcination to form pure phase oxide films. Two types of copper surfaces were investigated and compared: copper films deposited by the thermal evaporation (PVD method) and films obtained by electroplating. The electroplating method is a simple, low-cost process [42,43,44], while the PVD technique allows for the production of homogeneous metallic coatings with excellent adhesion [45]. The combination of both methods with the chemical growth of nanostructures and ammonolysis were tested to fabricate more topographically varied, three-dimensionally structured Cu_3_N films, that are impossible to obtain via the usually utilized magnetron sputtering method. During the research, a number of factors affecting the properties and morphology of the films were examined, such as: the fabrication method of primary copper substrates and conditions of growth. The described method of 3D Cu_3_N nanostructure fabrication, including the PVD step, provide an excellent choice leading to high surface area nitride layers that are thinner than 200 nm. Moreover, the studies presented by us cover an important current research area concerning the design of nanostructured films which, so far, has been rarely exploited for copper nitride.

## 2. Materials and Methods

### 2.1. Materials

CuSO_4_∙5H_2_O (POCh, Gliwice, Poland) and NH_3_∙H_2_O (25%, Chempur, Piekary Śląskie, Poland) were of analytical grade and used as purchased. Three-times distilled water was used for experiments.

### 2.2. Cu_3_N Thin Films on Electrodeposited Copper Surfaces

The electrochemical deposition of copper films was performed in a 0.5 M CuSO_4_∙5H_2_O electrolyte solution using silicon wafers as a cathode (1 × 3 cm, crystallographic orientation (111)) and copper plates (1 × 3 cm) as an anode. The silicon substrates were prepared (activated) by etching in a HF/HNO_3_/H_2_O mixture (1:4:5) or polishing with sandpaper of various grit sizes (1500, 2000, 3000, 7000). In a series of depositions, different electrolysis parameters, such as time (5 s–30 min), voltage (0.5–3.0 V) and current (0.01–0.11 A) were applied. Deposited copper surfaces were etched in 4 M HCl solution for 15 min and washed three times with distilled water. Then the prepared surfaces were placed in vessels with 15 mL of 0.033M or 0.066 M ammonia solution for 48, 72 or 96 h at ambient temperature. Then the vessels were covered with parafilm using two different approaches: a covered system and a partially-covered system (with holes in the parafilm). Afterwards, the samples were removed from the ammonia solution, gently washed three times with distilled water and allowed to dry. The obtained films were heated in a tube furnace in a two-step process: first, at 120 °C for 1 h, and then at 180 °C for 2 h. Ammonolysis experiments of the prepared oxide precursors were conducted in a horizontal tube furnace at 310 °C for 4 h.

### 2.3. Cu_3_N Thin Films on Copper Surfaces Deposited by Thermal Evaporation

Copper films with 30 nm thickness were fabricated by thermal evaporation in a vacuum on 1 × 1 cm silicon substrates (crystallographic orientation (111) or (100)). The base pressure was below 2∙10^−5^ Pa and the deposition rate was 1 Å/s. The nominal thickness (dn) and the evaporation rate (v) of the films were monitored by a quartz microbalance (6.9 MHz). The deposited Cu films were immersed in 0.033 M or 0.066 M ammonia solution for 3 to 24 h at room temperature. After the immersion process, the samples were removed from the solution, washed three times with distilled water and allowed to dry in the air. The obtained films were placed in a tube furnace and heated at 120 °C for 1 h and at 180 °C for 2 h. Ammonolysis experiments of the prepared oxide precursors were conducted at 310 °C for 4 h.

### 2.4. Characterization

The morphology of the obtained specimens was investigated by scanning electron microscopy (SEM) studies, performed with a Quanta 3D FEG (FEI, Hillsboro, OR, USA) (EHT = 30 kV) instrument. Films fabricated on a silicon substrate were placed onto carbon tabs attached to aluminum stubs and analyzed without coating treatment. Transmission electron microscopy (TEM), with selected area electron diffraction (SAED) measurements, was conducted on a carbon-coated copper grid using a Tecnai F20 X-Twin (FEI, Hillsboro, OR, USA) instrument. The transference process of the deposited structures was performed by gently placing and pressing TEM grids on the Si substrates. Phase identification was examined by powder X-ray diffraction (XRD) using an X’Pert Pro θ-2θ diffractometer (Malvern Panalytical Ltd, Malvern, UK) with CuKα radiation. The X-ray diffraction patterns were compared with reference card files by PowderCell v.2.3 software [26]. The UV–Vis spectra of samples were registered by diffuse reflectance spectroscopy (DRS) (V-750 UV–Visible Spectrophotometer, JASCO, Tokyo, Japan). The FT-IR analysis was carried out in the attenuated total reflectance (ATR) mode in the spectral range of 100–4000 cm^−1^ (FT-IR Vertex 70V spectrometer, Bruker Optik, Ettlingen, Germany). The thickness and optical constants of the prepared films was determined by spectroscopic ellipsometry (SE) using the V-VASE device (J. A. Woollam Co., Inc., Lincoln, NE, USA). The composition of the sample was investigated by X-ray photoelectron spectroscopy (XPS). The photoemission process was initialized by RS 40B1 Prevac (Rogów, Poland) Al Kα X-ray source (1486.6 eV) at an angle of 55 degrees with respect to the normal of the sample. The energy of photoelectrons was measured by using VG-Scienta R3000 (Uppsala, Sweden) hemispherical energy analyzer, the step of spectra was set to ΔE = 0.1 eV. Spectra were calibrated to adventitious carbon C 1s peak (284.8 eV). In order to perform a quantitative analysis, the experimental data were fitted to Gauss–Lorentz shapes using CasaXPS software (version 2.3.16, Casa Software Ltd., Teignmouth, UK).

## 3. Results and Discussion

### 3.1. Cu_3_N Thin Films on Electrodeposited Copper Surfaces

**Figure 1:**
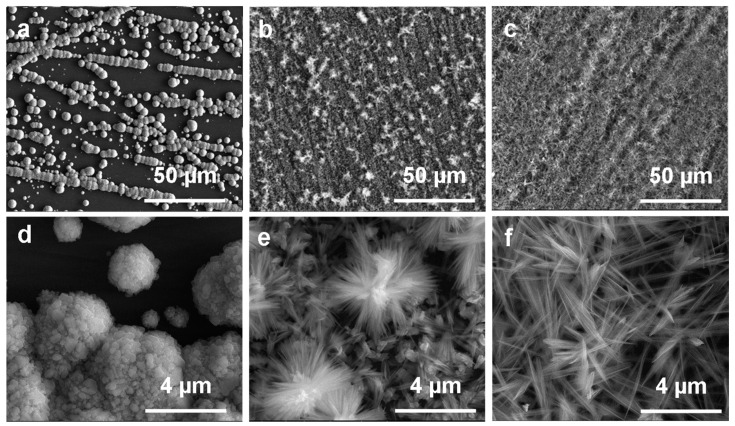
Scanning electron microscopy (SEM) images: (**a**,**d**) electrodeposited copper surfaces (3 V, 0.1 A, 30 s), (**b**,**e**) the same surfaces after immersion process in 0.033 M, and (**c**,**f**) in 0.066 M NH3 solution (48 h, partially-covered system).

Structures formed on the silicon wafer during the electro-deposition featured different morphologies and sizes depending on the substrate preparation and process conditions used. The initially tested etching of silicon surfaces by HF/HNO_3_ solutions resulted in the non-uniform coverage of the substrate with metallic copper. Thus, this activation mode of Si plates before electrodeposition was abandoned in favor of mechanical treatment. The technique of polishing the Si surface as well as the grit size of the sandpaper had a significant impact on the final layer topography. Rubbing the surface several times in one direction resulted in a uniformed distribution of copper crystallites on the substrate. Using the highest gradation sandpaper resulted in the formation of copper crystallites with the smallest diameter (*ca*. 2–4 μm), while for the lowest gradation, crystallites of *ca*. 5–10 μm diameter were observed. This effect of size reduction was also observed with decreasing time of the electrolysis from 15 min to 30 s. As a result of many pre-experiments, during which the proper sandpaper type (7000 grit) and electrolysis parameters (U = 3 V, I = 0.1 A, t = 30 s) were selected, copper particles with a diameter of 2–4 μm consisting of single 200–700 nm length grains were obtained (Figure 1a,d).

Conditions of the growth process in the ammonia solution highly affected the final shape of formed structures and the composition of films. Immersion of the Cu surface in the 0.033 M NH_3_ solution for 48 h resulted in the formation of flower-shaped micro-structures, consisting of 2–3 μm in length, needle-shaped nano-structures (Figure 1b,e). Increasing the solution concentration to 0.066 M contributed to the growth of longer (2–5 μm) and more densely distributed needle-shaped structures (Figure 1c,f). While extending the immersion time to 72 h led to complete dissolution (leaching) of the films. Moreover, changing the thermodynamic conditions of the process influenced the structures morphology as well—spherical structures with some composed of plate-like crystallites were observed for the samples placed in a covered system (Figure S1), while densely embedded needle-shaped structures were obtained in a partially-covered system (Figure S2).

**Figure 2:**
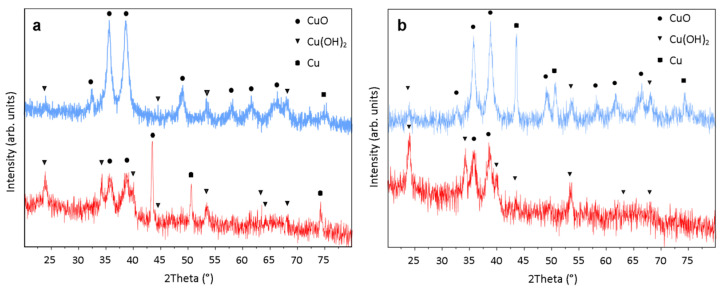
X-ray diffraction (XRD) patterns: (**a**) films grown in 0.033 M NH_3_ solution for 48 h in a partially-covered system (red) and in a covered system (blue), (**b**) films grown in 0.066 M NH_3_ solution for 48 h in a partially-covered system (red) and in a covered system (blue).

Powder X-ray diffraction studies indicated that films after the immersion process featured multiphase compositions. All registered diffractograms, both for the structures grown in 0.033 M (Figure 2a) and 0.066 M (Figure 2b) NH_3_ solutions, exhibited reflections characteristic of CuO, Cu(OH)_2_ and for metallic copper traces.

These results are consistent with the visual assessment of the films and solutions. The samples exhibiting high intensity of CuO reflections were black colored and the final solution pH was about 9–10, whereas the samples featuring high intensity of Cu(OH)_2_ signals were blue colored and the solution pH was about 11. Such a difference in the composition of both types of samples can be explained by the varied supply of oxygen to the system during the immersion process conjugated with gradual releasing of NH_3_ resulting in a change of pH value. The CuO rich samples were obtained in a covered vessel, whereas samples exhibiting Cu(OH)_2_ signals were obtained in a partially-covered system.

According to observed diffractograms and SEM images of both types of films, we found that the nano-needle Cu(OH)_2_ structures growth increases with increasing oxygen concentration in the system. X. Wen et al. reported similar considerations for the synthesis of Cu(OH)_2_ nanoribbon arrays from copper foils, explaining that the nanoribbon growth rate increased with the oxygen concentration [46]. The mechanism of growth is based on the dissolution of bare copper in ammonia containing solutions, which has been extensively studied by many authors [46,47,48,49]. The first step is the adsorption of dissolved oxygen on the copper surface, providing the Cu^2+^ ions. As a result, copper oxide can be formed as well. Subsequently, NH_3_ molecules react with copper ions resulting in copper ammine complex formation, and OH^-^ ions replace NH_3_ providing the growth of Cu(OH)_2_. The following reactions can be proposed, Equations (1) and (2):

H_2_O + Cu + ½O_2_ + 4NH_3_ → Cu(NH_3_)_4_^2+^ + 2OH^−^ (1)

Cu(NH_3_)_4_^2+^ + 2OH^−^ ↔ Cu(OH)_2_ + 4NH_3_ (2)

**Figure 3:**
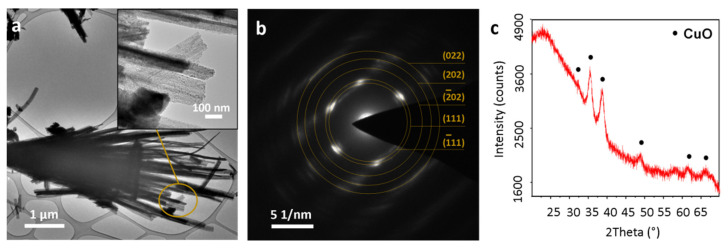
CuO needle-shaped structures detached from the substrate: (**a**) transmission electron microscopy (TEM) images, (**b**) selected area electron diffraction (SAED) pattern of the inset area, (**c**) XRD pattern of CuO film.

Changes in the film’s composition depend on the conditions of their growth and had a significant impact on the final shape of the structures. In order to obtain a homogenous CuO film, the samples were thermally treated, after which the final morphology and size of the structures remained unchanged. TEM images exhibit the polycrystalline needle-shaped structures with approximately 20–70 nm thickness and 2–6 μm length, indicating the unchanged morphology of the primary structures (Figure 3a).

Selected area diffraction pattern (Figure 3b) composed of several rings can be well assigned to the monoclinic copper(II) oxide. High intensity spots with d spacings values equal to 2.51 and 2.31 Å are associated with the (1$\bar{1}$1) and (111) planes. Weak intensity spots with d spacings of 1.85, 1.58 and 1.40 Å related to the (2$\bar{0}$2), (202) and (022) planes can also be distinguished. These results are in agreement with the obtained XRD pattern (Figure 3c). Characteristic reflections at 35.39°, 38.81°, 48.91°, 58.21°, 61.71° and 66.36° 2ϴ values, corresponding to (1$\bar{1}$1), (111), (2$\bar{0}$2), (202), (1$\bar{1}$3) and (022) planes of monoclinic CuO (C2/c space group) were recorded.

**Figure 4:**
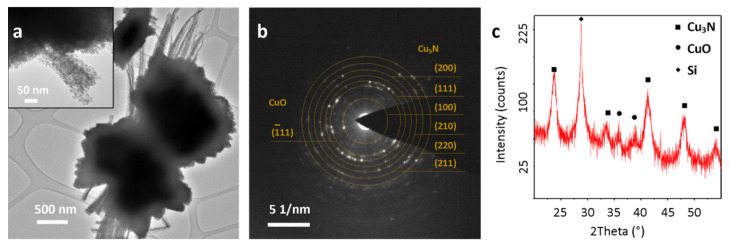
Cu_3_N structure detached from the substrate: (**a**) TEM images, (**b**) SAED pattern of the inset area, (**c**) XRD pattern of Cu_3_N layer.

The CuO film heated under gaseous ammonia flow was successfully transformed into Cu_3_N, and this was confirmed by TEM and XRD analysis. TEM analysis of the obtained Cu_3_N film showed that the ammonolysis reaction had no influence on the final morphology of the structures—needle-shaped copper nitride nanostructures were well preserved (Figure 4a).

SAED pattern (Figure 4b) showed the presence of anti-ReO_3_ Cu_3_N (Pm$\bar{3}$m space group) planes: (100), (111), (200), (210), (211), (220), corresponding to the calculated d spacings: 3.81, 2.20, 1.91, 1.69, 1.54 and 1.36 Å. High intensity spots with d spacings equal to 2.51 Å, characteristic for CuO (1$\bar{1}$1) plane were also observed. The XRD pattern indicated the presence of high intensity Cu_3_N reflections at 23.70°, 33.28°, 41.35°, 47.97° and 53.96° 2ϴ values, with a silicon peak from the substrate at 28.77° and weak CuO signals at 35.80° and 38.90° (Figure 4c).

### 3.2. Cu_3_N Thin Films on Copper Surfaces Deposited by Thermal Evaporation

The above-presented method for nano-structured copper nitride thin film fabrication is a new approach and can be used successfully for particular purposes. However, during our research, we noticed some limitations of this pathway, for example, the inability to obtain thinner or more uniform coatings. Despite the growth of nanowires from deposited metallic copper micro-lines, resulting in relatively good coverage of the entire surface of the substrate, we decided to modify the presented procedure. In order to avoid the above mentioned disadvantage, the physical deposition technique was applied to form initial metallic layers. This allowed us to proceed with the nanowire growing stage from a much thinner and more homogeneous metallic layer. Additionally, the PVD process is more controllable than the electrodeposition technique.

**Figure 5:**
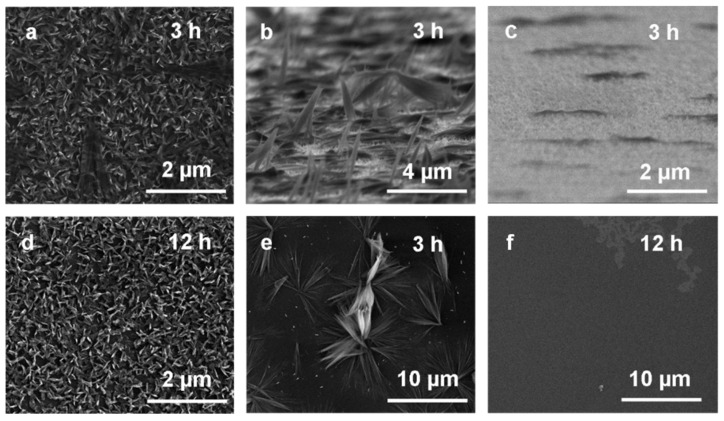
SEM images of copper surfaces immersed in 0.033 M ammonia solution: (**a**–**c**) for 3 h, (**d**) for 12 h. SEM images of copper surfaces immersed in 0.066 M ammonia solution: (**e**) for 3 h, (**f**) for 12 h.

Differences in the color of the films grown via the modified pathway were observed, viz. from blue–green to black, however, this phenomenon reflected the nano-scale of the samples, rather than changes in their chemical composition. Analysis of the SEM micrographs of layers immersed in 0.033 M ammonia solution for 3 h pointed to the formation of densely packed needle-like crystallites of less than 200 nm in length (see Figure 5a,c).

SEM images of a cross section view of some of the surfaces also indicated the presence of additional scattered single needle-shaped structures of 200–500 nm in width and 2–3 μm in length (Figure 5c). The growth of the longest needles could be a result of disturbances in the growing conditions. As the immersion time was increased, more densely embedded architectures were observed on the surface (Figure 5d). Increasing the ammonia concentration to 0.066 M resulted in the growth of longer (*ca.* 5 μm) structures (Figure 5e). Immersion of copper in 0.066 M NH_3_ solution for 12 h (Figure 5f) or more, contributed to the complete dissolution of the layer. The same phenomenon was observed for the electroplated copper immersed in ammonia for 72 h.

**Figure 6:**
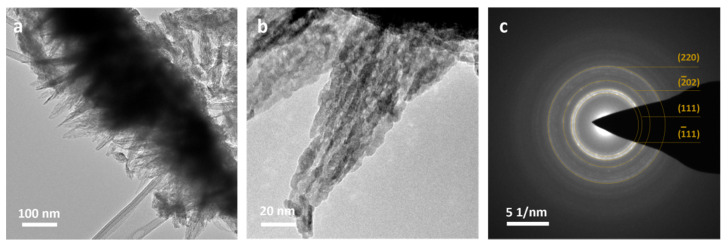
CuO needle-shaped structures grown in 0.033 M NH3 solution for 3 h and calcined: (**a**,**b**) TEM images, (**c**) SAED pattern of the inset area.

Due to the low thickness, and therefore small amount of material on the surface of the substrate, the grown films were difficult to characterize by XRD method. However, SAED analysis of the copper surface after immersion in ammonia indicated that the grown films consisted of CuO, and no characteristic spots or rings for Cu_2_O or Cu(OH)_2_ were observed. After thermal treatment up to 180 °C, the shape and size of structures were well-preserved (Figure 6a,b). The SAED pattern consisted of characteristic rings for copper(II) oxide (1$\bar{1}$1), (111), (2$\bar{0}$2) and (220) and planes, without any spots for Cu(OH)_2_ (Figure 6c).

**Figure 7:**
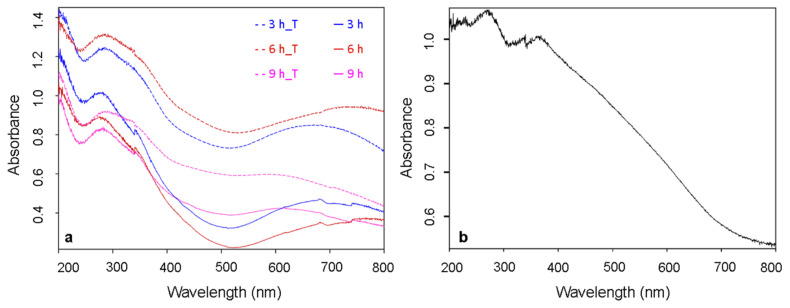
Absorption spectra of (**a**) copper surfaces immersed in 0.033 M ammonia solution for 3–9 h before and after thermal treatment (marked with the ‘T’ index); (**b**) Cu_3_N film.

These results did not comply with the visual assessment of the samples because the color of the samples did not change to black, as would be expected for CuO. Therefore, again, it can be suggested that the color phenomenon in the films prepared by PVD deposition is related to the nano-dimensions of the films rather than their properties. The absorption spectra of the samples grown in 0.033M ammonia solution for 3–9 h exhibit the broad band between 250–380 nm that is characteristic of CuO (Figure 7a). The second broad peak with a maximum above 600 nm could be the result of an interference effect associated with the relatively thick dielectric film and the different positions of the peaks maxima are observed for layers fabricated in various experimental conditions, affecting the morphology of the films. Absorbance spectra shown in Figure 7b exhibit the maxima characteristic for copper nitride (below 400 nm) [50].

The interference effect is screened, in this case, by the mentioned inter-band transitions. The detailed analysis of the optical properties of CuO and Cu_3_N films will be presented later in this section when discussing the SE results.

A similar shape of the absorption band was recorded by Y. Zhao et al., who synthesized 2D CuO nanoleaves from Cu(OH)_2_ nanowires. Their studies also confirmed that the absorption band parameters depend on the morphology of the CuO structures [51]. After thermal treatment, changes in the spectra of the same samples were not observed (Figure 7a).

Due to the fact that the registered DRS spectra contain information not only derived from the produced 3D oxide or nitride structures, but also from the substrate (Si/SiO_2_), the band gap (*E_g_*) of the CuO and Cu_3_N layers was determined by the ellipsometric method.

**Figure 8:**
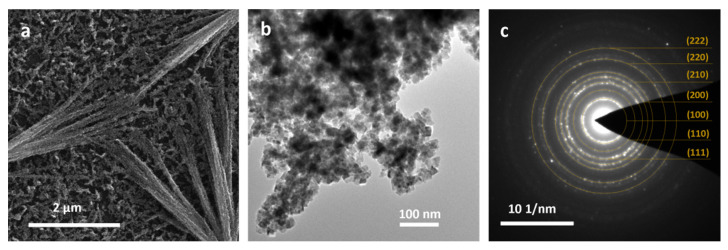
Cu_3_N nanocrystals: (**a**) SEM image, (**b**) TEM image, (**c**) SAED pattern.

For further experiments, samples obtained by immersion in 0.033 M solution for 3 h were selected, because they were the most homogeneous and reproducible. As a result of the ammonolysis reaction, the original needle-shaped structures retained their shape and size, however, the analysis of needles by TEM images indicated the nanocrystallites forms (Figure 8a,b).

SAED pattern with characteristic rings of (111), (110), (100), (200), (210), (220) and (222) planes confirmed formation of the copper nitride (Figure 8c). XRD pattern exhibits reflection with a small intensity—a characteristic of Cu_3_N at the 23.52° 2ϴ value—but the result was not confirmed due to the presence of residual reflections from silica substrate. According to the results from the diffuse reflectance spectroscopy, the Cu_3_N nanocrystals exhibit absorption over the entire spectrum range, with a maximum at 268 nm^−1^ (Figure 7b). FT-IR spectra registered in ATR mode (Figure S3) exhibited a sharp band at 615 cm^−1^, which is characteristic for Cu-N lattice vibration [52]. Additionally, a small signal was recorded at 1100 cm^−1^, assigned to Si-O-Si vibrations from the SiO_2_ layer covering the Si substrate [53].

**Figure 9:**
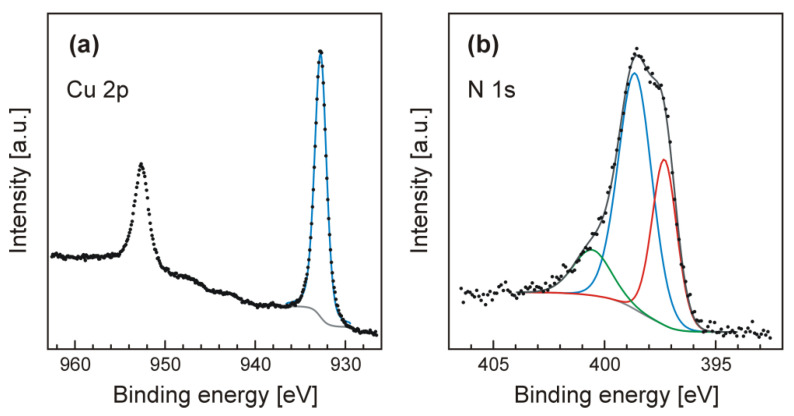
X-ray photoelectron spectroscopy (XPS) spectra: (**a**) Cu 2p core level, (**b**) N 1s level.

Figure 9 presents the XPS results obtained from the sample after formation of the Cu_3_N film.

The copper peaks shown in Figure 9a were detected at the energies of 932.7 eV (Cu 2p3/2) and 952.7 eV (Cu 2p1/2). Cu 2p3/2 level was easily fitted with a single peak, having a relatively small full width at half the maximum (FWHM) of 1.55 eV. The obtained spectrum of Cu differs significantly from the spectra observed for the CuO samples, in which case the strong satellite peaks are usually found at about 941 and 944 eV. Moreover, in CuO spectra both components of the Cu 2p doublet are usually broader, and are fitted with at least two peaks [54,55]. This is not the case in the nitride sample, which gives strong evidence that CuO is not present—at least not in the surface area. The nitrogen peak, presented in Figure 9b, consists of three components. The strongest, found at 398.6 eV is probably attributed to Cu_3_N. The second one, detected at 397.3 eV, can be assigned to N atoms dissolved into the Cu_3_N film. Moreover, there is also at least one low intensity peak noted at higher binding energies (400.6 eV) of uncertain origin. One possible hypothesis is that some nitrogen atoms were C, O or H bound when the sample was exposed to the ambient atmosphere prior to XPS measurements, and therefore, contaminants are present on the surface. Another suggestion is that it can be either a satellite peak, or from nitrogen atoms bound with the silicon substrate—forming NSiO_2_, because Si (*ca.* 5%) was also observed in XPS.

The complex refractive index, $\tilde{n}=n+ik$ (n is the real part of $\tilde{n}$ and *k* is the extinction coefficient), of the fabricated CuO and Cu_3_N films and their thicknesses were determined by spectroscopic ellipsometry. The ellipsometric azimuths, Ψ and Δ, were analyzed using a four-medium optical model of a sample: Si (substrate)\native SiO_2_ (2.5 nm)\CuO or Cu_3_N film\ambient. The complex refractive index of the substrate (Si and SiO_2_) was taken from the database of optical constants [56]. The 3D dielectric film (CuO or Cu_3_N) was considered as an effective medium (BEMA; Bruggeman Effective Medium Approximation) [56,57]. In this approach the complex refractive index of CuO or Cu_3_N was calculated using the following equation [56,57], Equation (3):

(3) $$f_{ambient}\frac{n_{ambient}^{2}-{\tilde{n}}_{effective}^{2}}{n_{ambient}^{2}+2{\tilde{n}}_{effective}^{2}}+(1-f_{ambient})\frac{{\tilde{n}}_{dielectric}^{2}-{\tilde{n}}_{effective}^{2}}{{\tilde{n}}_{dielectric}^{2}+2{\tilde{n}}_{effective}^{2}}=0$$

In Equation (3), $f_{ambient}$ is the fraction of ambient in the effective layer (the porous CuO or Cu_3_N film) and $n_{ambient}^{2}=1$ is the refractive index of air. The quantities ${\tilde{n}}_{effective}^{2}$ and ${\tilde{n}}_{dielectric}^{2}$ are complex refractive indexes of the effective (3D architecture; a mixture of dielectric and ambient) CuO (or Cu_3_N) film and CuO (or Cu_3_N), respectively. This approach allows us to extract the optical constants of copper oxide (or copper nitride), not the properties of the effective material. Moreover, we have estimated the concentration of ambient in the effective medium.

**Figure 10:**
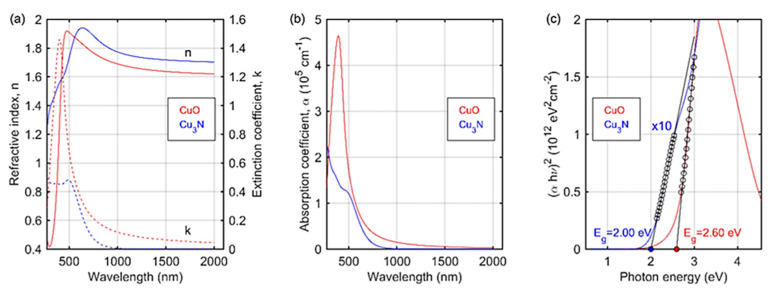
(**a**) The refractive index and the extinction coefficient, (**b**) absorption coefficient and (**c**) the Tauc plot for the CuO and Cu_3_N layers.

The determined thicknesses of the CuO and Cu_3_N layers were 176 ± 3 nm and 169 ± 4 nm, respectively. The estimated value of $f_{ambient}$ was 39 ± 4% for CuO and 40 ± 16% for Cu_3_N. It should be noted that both the obtained thickness and the fraction of ambient in the material are related to the dense CuO (or Cu_3_N) film, excluding the scattered, much longer needles (see Figure 6b). The optical constants of the CuO (or Cu_3_N) film were parameterized using a sum of Gaussian and Lorentzian oscillators. Mathematical formulas for these dispersion relations can be found in [56,57]. The complex refractive index and the extinction coefficient of CuO and Cu_3_N films is presented at Figure 10a.

The spectra exhibit semiconducting behaviors with the band-gap in the visible spectral range. Although the presence of air in the effective layer was considered, the extracted refractive index is smaller than the one obtained for the dense materials [50,58]. This result can be explained considering the structure of the layer. Apart from the voids between the crystallites (included as $f_{ambient}$ in Equation (3)), the synthesized films exhibit nanoporosity [59]. The absorption coefficient (Figure 10b) in the absorbing spectral range for CuO is two times larger, than that obtained for Cu_3_N. Moreover, the maximum of the alpha value for copper monoxide can be noted as 390 nm, while for copper nitride it arises outside the measuring spectral range. For both materials, the absorption coefficient significantly decreases to a value close to zero in the visible spectral range. To estimate the value of the band-gap energy, the Tauc method was used. In this approach (*αhν*)*^1/m^* is plotted as a function of the photon energy (*hν*), where *m* is a quantity related to the type of transition: *m* = 1/2 for direct allowed transition, *m* = 3/2 for direct forbidden transition, *m* = 2 for indirect allowed transition and *m* = 3 for indirect forbidden transition [60]. The determined alpha values at the level of 10^5^ cm^−1^ suggest the direct transition [61] (*m* = 1/2). The Tauc plot for the examined films is presented in Figure 10c. The estimated value of *E_g_* is 2.60 ± 0.04 eV (477 ± 7 nm) and 2.00 ± 0.01 eV (620 ± 3 nm) for CuO and Cu_3_N, respectively. The band-gap energy determined for Cu_3_N is comparable to [50,62] or larger [61,63] than the experimental results or theoretical calculations [21,64]. The literature value of the band-gap energy for CuO is in the range off 1.2 to 2.8 eV [65,66,67,68,69,70,71,72,73], however, the value obtained in this work is comparable to the values obtained for CuO nanoparticles [65,66,67,68,69,70,71,72,73,74].

## 4. Conclusions

In this study, copper nitride thin films were obtained by a facile solution-based method, starting from different copper surfaces: deposited by use of the PVD method (30 nm thickness) and by electroplating (2 μm thickness). The compositions and morphologies of the formed specimens were controlled by the conditions of the immersion of copper surfaces in ammonia solution. Immersion of electrodeposited copper films for 48 h in ammonia solution resulted in the formation of needle-shaped nanostructures of 2–3 μm long, whereas for the PVD obtained copper films, well-aligned nano-needles were obtained after 3–12 h. All electrodeposited films featured complex composition before thermal treatment—along with an increasing oxygen concentration during the growth process, the ratio of hydroxide to copper oxide increased. Thermal decomposition to pure CuO, and the ammonolysis reaction to Cu_3_N, had no impact on the final morphology and size all of the structures. For both types of starting copper films, polycrystalline needle-shaped Cu_3_N nanostructures were obtained.

The ammonolysis reaction allows one to obtain well-preserved in shape Cu_3_N structures, and thus, the CuO precursor seems to be a good choice for the fabrication of well-defined nanostructural materials. The electroplating/chemical growth is a simple combined method for obtaining these precursors. The subsequent heating of the as-prepared CuO in gaseous ammonia, is a convenient synthetic pathway leading to a micrometric Cu_3_N coating, especially when the high homogeneity of films is not a priority. For thinner, more uniform nanowire arrays, using the PVD method for the initial copper layer deposition is a more appropriate solution.

The synthesis of the presented copper nitride thin films, by applying the combined technique, extend the methodology of this semiconductor-based material fabrication. The large surface area of the synthesized nanostructures could be considered as being particularly important for potential applications as a catalyst. Moreover, the structures that have been achieved via our approaches can be easily modified by adding the polymer or subsequent deposition of another material, leading to new composite materials.

## Data Availability

The data presented in this study are available on request from corresponding author.

## Supplementary Materials

The following are available online at https://www.mdpi.com/1996-1944/14/3/603/s1, Figure S1: SEM images of electrodeposited copper surfaces (3V, 0.1 A, 30 s) (a) after immersion process in 0.033 M (a–b), and in 0.066 M NH_3_ solution (c–d) (48 h, covered system), Figure S2: SEM images of electrodeposited copper surfaces (3V, 0.1 A, 30 s) (a) after immersion process in 0.033 M (a), and in 0.066 M NH_3_ solution (b) (48 h, partially-covered system), Figure S3: FT-IR (ATR) spectrum of Cu_3_N film.
