# Supplementary material for: Copper Nitride Nanowire Arrays—Comparison of Synthetic Approaches
Source: Materials (Basel). 2021 Jan 28;14(3):603. doi: 10.3390/ma14030603 (PMC7865675; doi:10.3390/ma14030603)
Supplement: Supplementary file 1 [file materials-14-00603-s001.pdf]

materials

Supplementary Information

Copper Nitride Nanowire Arrays—Comparison of Synthetic Approaches

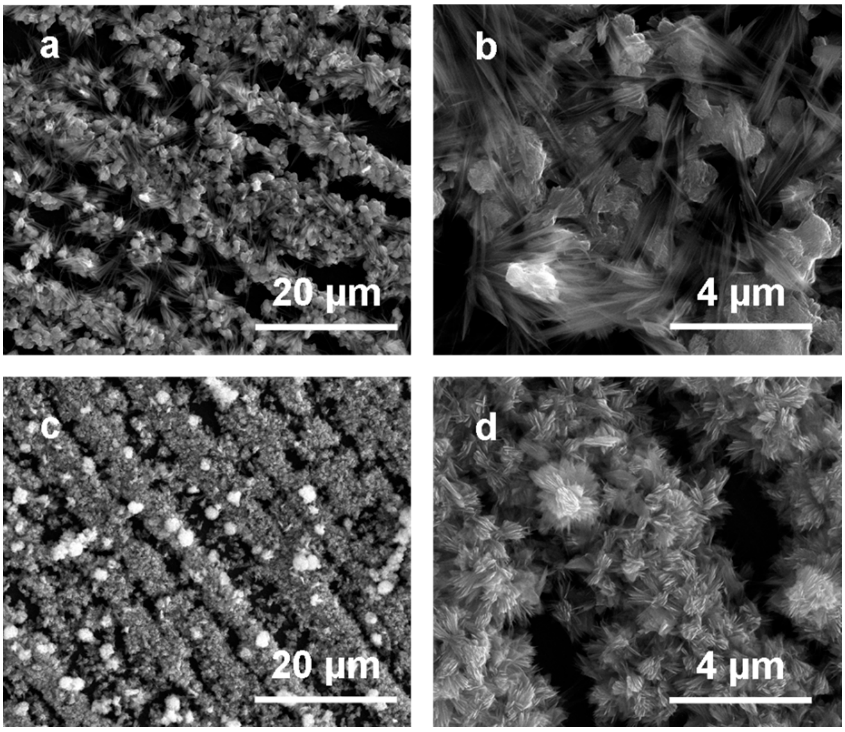

Figure 1. Scanning electron microscopy (SEM) images of electrodeposited copper surfaces (3V, 0.1 A, 30 s) (**a**) after immersion process in 0.033 M (**a**–**b**), and in 0.066 M NH_3 solution (**c**–**d**) (48 h, covered system).

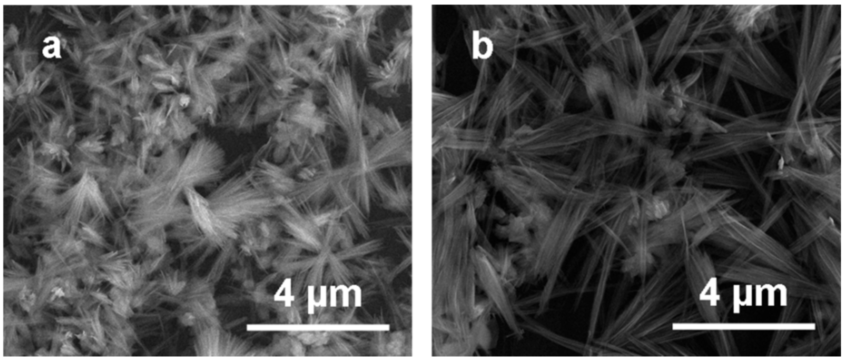

Figure 2. Scanning electron microscopy (SEM) images of electrodeposited copper surfaces (3V, 0.1 A, 30 s) (**a**) after immersion process in 0.033 M (**a**), and in 0.066 M NH_3 solution (**b**) (48 h, partially-covered system).

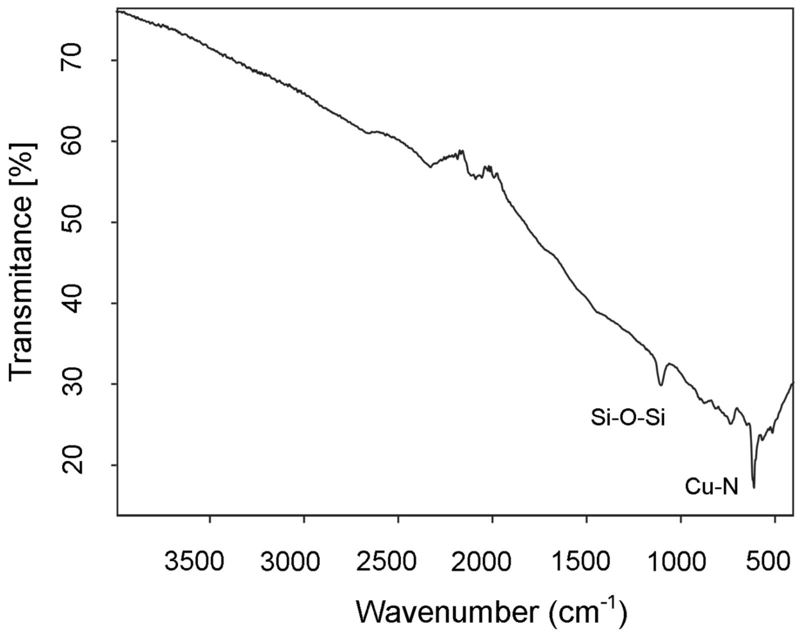

Figure 3. FT-IR (ATR) spectrum of Cu_3N film.
